# Bias due to participant overlap in two‐sample Mendelian randomization

**DOI:** 10.1002/gepi.21998

**Published:** 2016-09-14

**Authors:** Stephen Burgess, Neil M. Davies, Simon G. Thompson

**Affiliations:** ^1^ Department of Public Health and Primary Care, University of CambridgeCambridgeUK; ^2^ MRC Integrative Epidemiology Unit, University of BristolBristolUK; ^3^ School of Social and Community Medicine, University of BristolBristolUK

**Keywords:** aggregated data, instrumental variables, Mendelian randomization, summarized data, weak instrument bias

## Abstract

Mendelian randomization analyses are often performed using summarized data. The causal estimate from a one‐sample analysis (in which data are taken from a single data source) with weak instrumental variables is biased in the direction of the observational association between the risk factor and outcome, whereas the estimate from a two‐sample analysis (in which data on the risk factor and outcome are taken from non‐overlapping datasets) is less biased and any bias is in the direction of the null. When using genetic consortia that have partially overlapping sets of participants, the direction and extent of bias are uncertain. In this paper, we perform simulation studies to investigate the magnitude of bias and Type 1 error rate inflation arising from sample overlap. We consider both a continuous outcome and a case‐control setting with a binary outcome. For a continuous outcome, bias due to sample overlap is a linear function of the proportion of overlap between the samples. So, in the case of a null causal effect, if the relative bias of the one‐sample instrumental variable estimate is 10% (corresponding to an *F* parameter of 10), then the relative bias with 50% sample overlap is 5%, and with 30% sample overlap is 3%. In a case‐control setting, if risk factor measurements are only included for the control participants, unbiased estimates are obtained even in a one‐sample setting. However, if risk factor data on both control and case participants are used, then bias is similar with a binary outcome as with a continuous outcome. Consortia releasing publicly available data on the associations of genetic variants with continuous risk factors should provide estimates that exclude case participants from case‐control samples.

## 1 INTRODUCTION

Mendelian randomization is the use of genetic variants as instrumental variables to assess and estimate the causal effect of a risk factor on an outcome from observational data (Davey Smith & Ebrahim, 2003; Burgess & Thompson, 2015). A recent methodological development in Mendelian randomization is the use of summarized data on associations of genetic variants with the risk factor and with the outcome to obtain causal effect estimates (Johnson, 2013; Burgess, Butterworth, & Thompson, 2013). These summarized data comprise the associations of the individual genetic variants with the risk factor and with the outcome taken from univariable regression analyses (beta‐coefficients and standard errors from linear or logistic regression as appropriate). Suitable summarized data for such analyses have been made publicly available for hundreds of thousands of genetic variants by some large consortia (Burgess et al., 2015a). Examples include associations of genetic variants with lipid fractions from the Global Lipids Genetics Consortium (The Global Lipids Genetics Consortium, 2013) and with type 2 diabetes from the DIAGRAM consortium (Morris et al., 2012). Mendelian randomization analyses using summarized data have suggested causal effects of adiponectin on type 2 diabetes risk (Dastani et al., 2012), insulin levels on endometrial cancer risk (Nead et al., 2015), and telomere length on risk of lung adenocarcinoma (Zhang et al., 2015).

The validity of a Mendelian randomization investigation depends on the instrumental variable (IV) assumptions being satisfied for all genetic variants (Lawlor, Harbord, Sterne, Timpson, & Davey Smith, 2008). In particular, any genetic variant used as an IV is assumed to be:

1. Associated with the risk factor;
2. Independent of confounders of the risk factor–outcome association; and
3. Independent of the outcome conditional on the risk factor and confounders of the risk factor–outcome association (Greenland, 2000; Sussman, Wood, & Hayward, 2010).

In this paper, we assume that the IV assumptions are satisfied for all genetic variants in the analysis. Variants can be weak (i.e., they do not explain much variation in the risk factor), but they are all assumed to be valid IVs.

Although estimates from IV analysis in a one‐sample setting (that is, genetic variants, risk factor and outcome are all measured in the same participants) are asymptotically unbiased, they can have substantial bias in finite samples (Stock, Wright, & Yogo, 2002; Burgess & Thompson, 2011). This bias (known as weak instrument bias) acts in the direction of the confounded observational association between the risk factor and outcome (Nelson & Startz, 1990; Bound, Jaeger, & Baker, 1995). Its magnitude depends on the strength of association between the IV(s) and the risk factor (Staiger & Stock, 1997). Weak instrument bias also leads to inflated Type 1 error rates (over‐rejection of the null) (Stock & Yogo, 2002). One way of combatting weak instrument bias in practice is a two‐sample analysis strategy, in which the associations of IVs with the risk factor and with the outcome are obtained from two non‐overlapping datasets (Angrist & Krueger, 1992; Inoue & Solon, 2010). In a two‐sample Mendelian randomization analysis, any bias due to weak instruments is in the direction of the null (Pierce & Burgess, 2013). Bias in the direction of the null is less serious than bias in the direction of the observational association, as it is conservative, and so will not lead to inflated Type 1 error rates and false‐positive findings. This bias may lead to lower power to detect a causal effect and increased probability of a Type 2 error, although the standard errors typically also attenuate, mitigating this somewhat (Burgess, Dudbridge, & Thompson, 2016b).

The use of summarized data in Mendelian randomization is motivated by the increasing availability of suitable data in large sample sizes. A fortuitous side‐effect is that genetic associations with the risk factor and with the outcome are often obtained from separate datasets, leading to a two‐sample IV analysis. However, due to the nature of major international genetics consortia, often the datasets are not completely disjoint, and some studies and participants are in common between the two datasets. When there is some overlap, it is unclear whether bias due to weak instruments would be in the direction of the null (as in the case of zero overlap) or in the direction of the observational association (as in the case of complete overlap). While the sliding scale of bias toward the observational association as the proportion of overlap increases has previously been demonstrated (Pierce & Burgess, 2013), it is unclear in specific investigations whether this bias should be of concern.

The aim of this paper is to investigate the direction and degree of bias in a “two‐sample” instrumental variable analysis in which there is overlap between the two samples. This is achieved by theoretical considerations and a series of simulation studies with realistic parameters for Mendelian randomization. The structure of the paper is as follows. First, we explain why weak instrument bias occurs and the reason for the direction of the bias (Section 2). Next, we present simulation studies and discuss their results (Section 3 for a continuous outcome, Section 4 for a binary outcome). We present illustrations of the potential bias due to participant overlap in example Mendelian randomization investigations using summarized data from two large consortia (Section 5). Finally, we discuss the wider relevance of these results, and in particular the case of a two‐sample investigation in which the genetic variants were discovered in one of the datasets under analysis (Section 6).

## 2 WEAK INSTRUMENT BIAS

We initially assume that the outcome is continuous and that all regression analyses use a linear model. In the simplest case with a single IV, the causal effect of the risk factor on the outcome can be estimated as a ratio of regression coefficients (Martens, Pestman, de Boer, Belitser, & Klungel, 2006). The ratio estimate is the coefficient from regression of the outcome on the IV divided by the coefficient from regression of the risk factor on the IV (Didelez & Sheehan, 2007). In a one‐sample setting, weak instrument bias arises due to correlation between the regression coefficients in the numerator and denominator as a result of confounding between the risk factor and outcome (Nelson & Startz, 1990). In a two‐sample setting, the numerator and denominator in the ratio method will be uncorrelated. Bias with a single IV is difficult to quantify, as the expected value of the ratio estimate is undefined, because of the small but finite probability that the denominator in the ratio estimator (the IV–risk factor association) is arbitrarily close to zero (Hahn, Hausman, & Kuersteiner, 2004). However, if the IV–risk factor association is close to zero, then an IV analysis is unlikely to be performed in practice, as the first IV assumption (the only one that can be tested directly) appears to be violated. In simulation studies, the median ratio estimate across simulations is usually close to the true value of the causal effect even with complete sample overlap (a one‐sample analysis) except in the case of extremely weak instruments (those for which the expected strength of association with the risk factor corresponds to a *P*‐value above 0.05 Burgess & Thompson, 2011), indicating that the practical consequences of bias with a single IV are unlikely to be substantial (Angrist & Pischke, 2009).

### 2.1 Two‐stage least squares method

When there are multiple IVs, the two‐stage least squares (2SLS) estimate can be obtained in a one‐sample setting with individual‐level data by (i) regressing the risk factor on the IVs (first‐stage regression), and then (ii) regressing the outcome on fitted values of the risk factor from the first‐stage regression (second‐stage regression). Weak instrument bias can be explained as arising from overfitting in the first‐stage regression model, which occurs at least in part due to chance correlations of the IVs with confounders (Burgess & Thompson, 2011). In a one‐sample setting, the fitted values from the first‐stage regression are therefore correlated with the outcome in finite samples even in the absence of a causal effect. This leads to finite‐sample bias of the 2SLS estimate. The expected magnitude of this bias depends on the strength of association between the IVs and the risk factor through the concentration parameter. The concentration parameter (μ) is related to the expected value of the F statistic from the regression of the risk factor on the IVs: for large samples, $E\left(F\right)=\frac{\mu }{K}+1$, where *K* is the number of IVs (Cragg & Donald, 1993). We refer to the expected value of this *F* statistic as the *F* parameter, to emphasize that this is a characteristic of the population, and not simply a function of the observed data.

In a two‐sample setting with individual‐level data, the 2SLS estimate can be calculated by obtaining estimates of the first‐stage regression parameters in one dataset, and constructing fitted values of the risk factor in the second dataset using these estimates and the values of the IVs in the second dataset (measurements of the risk factor in the second dataset are not required). The outcome and the fitted values of the risk factor in the second‐stage regression are no longer correlated due to confounding. This approach is also known as split‐sample 2SLS (Angrist & Krueger, 1995). The fitted values of the risk factor are also equivalent (up to an additive constant) to values of an externally weighted allele score (an analysis approach used in Mendelian randomization (Burgess & Thompson, 2013)) in the second dataset using the first sample to obtain the external weights. Any bias due to weak instruments is in the direction of the null (Pierce & Burgess, 2013; Burgess et al., 2016b). This arises for the same reason as regression dilution bias in observational studies (Frost & Thompson, 2000). If the fitted values of the risk factor are imprecisely estimated (as will be the case with weak instruments), then they will suffer from non‐differential measurement error, and bias in the second‐stage regression will be in the direction of the null.

### 2.2 Inverse‐variance weighted method and equivalence to 2SLS method

If individual‐level data are not available, but instead summarized data on the associations of the genetic variants with the risk factor and with the outcome, then the 2SLS method cannot be implemented. Instead, an inverse‐variance weighted (IVW) method is often employed that combines the ratio estimates calculated separately for each IV, using a formula for performing a fixed‐effect meta‐analysis (Burgess et al., 2013). This method assumes that the IVs are uncorrelated (i.e., not in linkage disequilibrium), although extensions for correlated genetic variants have been proposed (Burgess et al., 2016b). The estimate from the IVW method is equal to the estimate from the 2SLS method asymptotically. The two estimates are also equal in finite samples when the correlations between the IVs are exactly zero (Burgess et al., 2016b). The level of weak instrument bias in the IVW method has been shown to be the same as that from the 2SLS method in realistic simulations (Burgess et al., 2013). We therefore expect the results of this paper to apply equally to analyses performed using the 2SLS method and individual‐level data, as to using the IVW method and summarized data.

Although we use the 2SLS method in some simulation studies of this paper for computational convenience, and we refer to theoretical results for weak instrument bias derived using the 2SLS method, the focus of this paper is the summarized data setting. Results from the 2SLS method are presented because of their similarity with those from the IVW method that can be performed using summarized data. If individual‐level data were available, then several alternative approaches for mitigating weak instrument bias would be possible, such as using either the limited information maximum likelihood (LIML) or the continuously updating estimator (CUE) method (Davies et al., 2015), using a jackknife IV estimator (Angrist et al., 1999) (or equivalently an allele score approach using leave‐one‐out cross‐validated weights Burgess & Thompson, 2013), or using an allele score approach using equal or externally specified weights (Burgess & Thompson, 2013). However, with summarized data, only the final option (equal or externally specified weights) is possible (Burgess et al., 2016b).

### 2.3 Magnitude of bias in a one‐sample setting

The ordinary least squares (OLS, also known as standard least squares regression) estimate is obtained by regressing the outcome on the risk factor. This “observational” estimate is typically biased due to confounding. The relative bias of the 2SLS estimate —the bias of the 2SLS estimate divided by the bias of the OLS estimate—is approximately and asymptotically equal to 1/E(F) (Staiger & Stock, 1997), where E(F) is the “F parameter.” An F parameter of 10 therefore corresponds to a 1/10=10% relative bias of the 2SLS estimate compared to the OLS estimate. However, this calculation cannot be directly employed for bias correction in an applied setting, as the F statistic in a given dataset may differ substantially from the F parameter due to random variation (Burgess, Thompson, & CRP CHD Genetics Collaboration, 2011). As weak instrument bias occurs due to chance correlations with confounders, the reference OLS estimate should ideally be unadjusted for measured confounders, unless these confounders are also adjusted for in the IV estimate.

### 2.4 Expected bias of 2SLS estimator

The bias of the 2SLS estimator in a one‐sample setting has been considered theoretically (Nagar, 1959). One approximation to the bias is:

(1) Bias  of 2 SLS  estimator ( one  sample )=σXY(K−2)σX2μ≈σXYσX2E(F)

where μ is the concentration parameter, *K* is the number of IVs, $\sigma_{X}^{2}$ is the variance of the error in the first‐stage regression model, and $\sigma_{XY}$ is the covariance of the error terms in the first‐ and second‐stage regression models (Bun & Windmeijer, 2011). In a two‐sample setting, this formula may not be directly applicable, as the sample sizes for the first‐ and second‐stage regressions (and so the lengths of the error term vectors) may differ, in which case a covariance cannot be calculated. If the sample sizes are equal, then we can decompose the covariance into a term corresponding to individuals included in both regressions, and a term corresponding to unrelated individuals that will have expectation zero. If the sample sizes differ, dependence between these two error terms is still driven by individuals in common between these two regressions, and the presence of individuals in only one or other of the regressions will dilute this dependence. As covariance is a linear operator in both its arguments, we may therefore expect the bias of the 2SLS estimator to be approximately linear in the proportion of overlap between the samples.

## 3 SIMULATION STUDY—CONTINUOUS OUTCOME

To investigate the degree and direction of bias in a “two‐sample” Mendelian randomization investigation where there is overlap between the samples, we conduct a simulation study. The data‐generating model is given below.

(2) gik∼ Binomial (2,0.3) independently  for k=1,...,20xi=∑k=120αgik+ui+εXiyi=βXxi+βUui+εYiui∼N(0,1),εXi∼N(0,1),εYi∼N(0,1) independently

The 20 IVs ($g_{ik},k=1,...,20$) are indexed by *k* and individuals are indexed by *i*. The IVs are modeled as independently distributed biallelic single nucleotide polymorphisms (SNPs) with a minor allele frequency of 0.3. The risk factor ($x_{i}$) is a linear function of the IVs, a confounder ($u_{i}$), and an independent error term ($\varepsilon_{Xi}$). The outcome ($y_{i}$) is a linear function of the risk factor, confounder, and another independent error term ($\varepsilon_{Yi}$). The IVs all have the same per allele effect on the risk factor (α). The causal effect of the risk factor on the outcome is $\beta_{X}$, and the effect of the confounder on the outcome is $\beta_{U}$.

We consider the cases of a positive causal effect ($\beta_{X}=0.2$) and a null causal effect ($\beta_{X}=0$), and take three values of the confounder effect on the outcome ($\beta_{U}=0.6,1,2$). The IV strength is varied by taking three values of α=0.04,0.06,0.08. We consider cases where there is 0% overlap, increasing in increments of 10% up to a 100% overlap. This is achieved by simulating data on 20, 000 individuals. Estimates were obtained using the 2SLS and IVW methods. The first‐stage regression is undertaken (or for the IVW method, IV–risk factor associations are estimated) in the first 10, 000 individuals, and the second‐stage regression (IV–outcome associations) in individuals 10, 001–20, 000 (0% overlap), individuals 9,001–19, 000 (10% overlap), individuals 8,001–18, 000 (20% overlap), and so on. Hence all associations were estimated using 10, 000 individuals. Total 10, 000 simulations were considered for each set of parameters.

Although some aspects of the simulation study are not varied here, we repeated the simulation study varying the number of IVs, and the total sample size (results not shown). In each case, the amount of bias was almost identical between scenarios in which the F parameter (expected value of the F statistic) was similar. Hence, we would expect the results of this simulation study to be generalizable to other situations, and would view the F parameter (which depends on the sample size, number of IVs, and the proportion of variance in the risk factor explained by the IVs) as the key measure of bias.

### 3.1 Results

**Figure 1:**
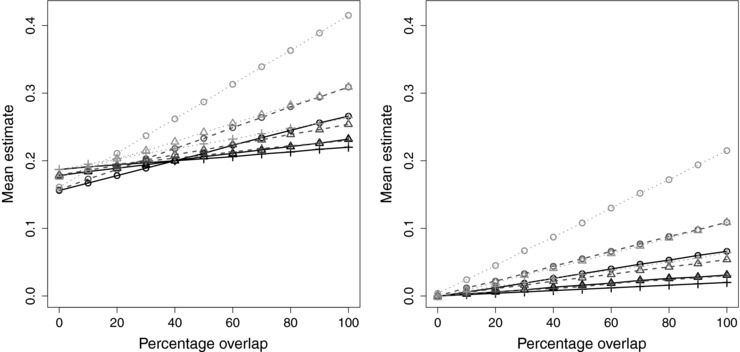
Mean two‐stage least squares/inverse‐variance weighted estimates plotted against sample overlap for different values of instrument strength (α=0.4, circle; α=0.6, triangle; α=0.8, plus) and different values of the confounder effect on the outcome ($\beta_{U}=0.6$, black solid line; $\beta_{U}=1$, mid‐gray dashed line; $\beta_{U}=2$, light‐gray dotted line). Left panel: positive causal effect ($\beta_{X}=0.2$); right panel: null causal effect ($\beta_{X}=0$)

Results are given in Table 1 and displayed visually in Figure 1. For each set of parameters, the mean estimate from the 2SLS method across simulations is given. Mean estimates from the IVW method were equal to those from the 2SLS method to 3 decimal places, and median estimates across simulations from both methods were similar to mean estimates. The Monte Carlo standard error for the mean estimates is 0.002 or less in all scenarios. Mean F and *R* ^2^ (coefficient of determination) statistics from regression of the risk factor on the IVs based on 10, 000 participants are provided to judge the strength of the instrumental variants. Ordinary least squares (OLS) estimates are also provided to help judge the level and direction of confounding.

**Table 1:** Simulation 1 with continuous outcome and different overlap proportions

	Mean	Mean	Percentage	$\beta_{X}=0.2$			$\beta_{X}=0$		
α	*F*	*R* ^2^	overlap	$\beta_{U}=0.6$	$\beta_{U}=1$	$\beta_{U}=2$	$\beta_{U}=0.6$	$\beta_{U}=1$	$\beta_{U}=2$
0.04	4.4	0.9%	OLS	0.498	0.697	1.193	0.298	0.497	0.993
			0%	0.156	0.157	0.161	−0.001	0.000	0.004
			10%	0.167	0.173	0.186	0.006	0.012	0.024
			20%	0.178	0.187	0.211	0.012	0.022	0.045
			30%	0.189	0.203	0.237	0.019	0.033	0.067
			40%	0.200	0.218	0.262	0.026	0.044	0.087
			50%	0.211	0.233	0.287	0.033	0.055	0.108
			60%	0.223	0.249	0.313	0.040	0.066	0.130
			70%	0.234	0.264	0.339	0.047	0.077	0.152
			80%	0.245	0.280	0.363	0.053	0.088	0.172
			90%	0.256	0.294	0.389	0.060	0.098	0.194
			100%	0.266	0.309	0.415	0.066	0.109	0.215
0.06	8.5	1.7%	OLS	0.495	0.692	1.185	0.295	0.492	0.985
			0%	0.178	0.179	0.176	0.000	0.000	−0.002
			10%	0.184	0.186	0.189	0.003	0.005	0.009
			20%	0.189	0.193	0.203	0.006	0.011	0.020
			30%	0.194	0.201	0.215	0.009	0.016	0.031
			40%	0.200	0.209	0.228	0.013	0.022	0.041
			50%	0.206	0.216	0.242	0.016	0.027	0.052
			60%	0.211	0.224	0.255	0.019	0.032	0.063
			70%	0.216	0.231	0.268	0.023	0.038	0.074
			80%	0.221	0.239	0.282	0.026	0.043	0.086
			90%	0.226	0.246	0.295	0.028	0.048	0.097
			100%	0.232	0.254	0.309	0.031	0.054	0.109
0.08	14.4	2.8%	OLS	0.492	0.687	1.174	0.292	0.487	0.974
			0%	0.187	0.187	0.187	0.000	0.000	0.000
			10%	0.191	0.191	0.195	0.002	0.003	0.007
			20%	0.194	0.195	0.203	0.004	0.006	0.014
			30%	0.197	0.199	0.211	0.006	0.009	0.020
			40%	0.200	0.204	0.218	0.008	0.011	0.026
			50%	0.203	0.208	0.225	0.010	0.014	0.032
			60%	0.206	0.213	0.232	0.012	0.018	0.038
			70%	0.210	0.218	0.240	0.014	0.022	0.044
			80%	0.213	0.222	0.248	0.016	0.024	0.050
			90%	0.217	0.226	0.255	0.018	0.027	0.056
			100%	0.220	0.230	0.264	0.020	0.030	0.064

Notes: Mean two‐stage least squares (or equivalently, inverse‐variance weighted) estimates with true causal effect $\beta_{X}=0.2$ (positive effect) and $\beta_{X}=0$ (null effect) for three values of genetic associations with the risk factor (α), three values of the confounder effect on the outcome ($\beta_{U}$), and 11 values of the percentage overlap between the two samples. The mean *F* statistic (*F*), mean coefficient of determination (*R* ^2^), and mean ordinary least squares (OLS) estimate are given to judge the strength of the instrumental variables and the degree of confounding.

With a positive causal effect, the results demonstrate clearly the transition from bias in the direction of the null with no overlap, to bias in the direction of the confounded association with increasing overlap. This transition happens earlier when the confounding is stronger, although the balance point where the biases cancel out does not seem to depend on the strength of the IVs: for $\beta_{U}=0.6$, it is around 40%; for $\beta_{U}=1$, it is around 28%; and $\beta_{U}=2$, it is around 16%. Further simulations (not shown) suggest that the balance point also depends on the magnitude of the causal effect, so the precise balance points in this simulation will not necessarily hold in other cases. With a null causal effect, there is no bias with 0% overlap, and bias increases as the degree of overlap increases.

### 3.2 Deriving analytic formulae for the expected bias under the null and type 1 error rate

The relationship between the level of overlap and the mean estimate appears to be linear throughout, both with a positive and with a null causal effect. Equally, with a null causal effect, the bias is proportional to the effect of the confounder ($\beta_{U}$), which in turn is proportional to the OLS estimate. This suggests that a formula can be derived for the amount of bias expected under the null hypothesis:

(3) Bias  under  null = OLS  estimate × Percentage  overlap 100× Relative  bias

where the relative bias is the reciprocal of the F parameter.

Given the bias and the standard error of an estimate, the expected Type 1 (false positive) error rate for a two‐sided test of size 5% can be approximated analytically as:

(4) Type 1 error  rate =2−Φ1.96− bias  standard  error −Φ1.96+ bias  standard  error

where Φ is the cumulative standard normal function. The variance of the IV estimate (the 2SLS or the IVW estimate) can be approximated as:

(5) $$\mathrm{VarianceofIVestimate}≃\frac{\mathrm{var}(R_{Y})}{N\;\mathrm{var}\left(X\right)\;\rho^{2}}$$

where *N* is the sample size for the IV–outcome association, $R_{Y}$ is the residual outcome after subtracting the causal effect of the risk factor ($R_{Y}=Y-{\hat{\beta }}_{X}X$), and ρ is the correlation between the IVs and the risk factor (the *R* ^2^ statistic – the proportion of variance in the risk factor explained by the IVs – is an estimate of ρ^2^) (Nelson & Startz, 1990). The variance of $R_{Y}$ is equal to the variance of *Y* when the causal effect of the risk factor on the outcome is zero. The relationship between the F statistic and the *R* ^2^ statistic (Dobson, 2001) is:

(6) $$F=\frac{N-K-1}{K}\;\frac{R^{2}}{1-R^{2}}$$

where *K* is the number of IVs. For small values of *R* ^2^, this means that the F and *R* ^2^ statistics are approximately linearly related.

Hence, given the sample size, sample overlap percentage, OLS estimate (in standard deviation units for the risk factor and outcome, otherwise the standard deviations of risk factor and outcome are required), and an estimate of the strength of the IVs (either the F statistic or the *R* ^2^ statistic), the bias and Type 1 error rate can be calculated. R code for performing these calculations is given in Web Appendix A1.

### 3.3 Validating the analytic formulae

To assess the validity of the analytic formulae for the bias and Type 1 error rate, we conducted a further set of 10, 000 simulations in 10, 000 participants using model (2) under the causal null ($\beta_{X}=0$), with the strongest level of confounding ($\beta_{U}=2$) and 100% sample overlap, with a wider range of values for the IV strength (α=0.01,0.02,...,0.1,0.15,0.2) to estimate the relative bias and empirical Type 1 error rate (proportion of simulations for which the Wald test for the IV estimate rejected the null) in each case.

These results are given in Table 2. The relative bias estimates are close to 1/E(F), as theoretically predicted (Staiger & Stock, 1997). The expected Type 1 error rate was calculated using the mean values of the *F* statistic, *R* ^2^ statistic, variance of the risk factor and outcome, and the OLS estimate across simulations. The empirical and expected Type 1 error rates showed fairly close agreement, with the expected Type 1 error rate tending to be a slight underestimate of the empirical rate for all but the weakest of instruments. The Monte Carlo standard error for the empirical Type 1 error rate was around 0.2%.

**Table 2:** Simulation 2 with continuous outcome to validate bias and type 1 error rate formulae

			Mean	Mean	Relative		Empirical	Expected
α	Mean *F*	Mean *R* ^2^	OLS estimate	IV estimate	bias	(Mean *F*)^−1^	Type 1 error	Type 1 error
0.01	1.2	0.2%	0.999	0.829	0.829	0.825	84.8%	89.5%
0.02	1.8	0.4%	0.998	0.531	0.532	0.545	65.5%	69.8%
0.03	2.9	0.6%	0.996	0.333	0.334	0.346	46.9%	46.1%
0.04	4.4	0.9%	0.993	0.216	0.218	0.229	32.5%	30.6%
0.05	6.3	1.2%	0.990	0.149	0.151	0.160	24.1%	21.8%
0.06	8.6	1.7%	0.985	0.109	0.111	0.117	19.2%	16.8%
0.07	11.3	2.2%	0.980	0.082	0.083	0.089	15.6%	13.6%
0.08	14.4	2.8%	0.974	0.062	0.064	0.069	12.4%	11.5%
0.09	18.0	3.5%	0.967	0.050	0.052	0.056	11.0%	10.1%
0.10	22.0	4.2%	0.960	0.040	0.042	0.046	9.8%	9.0%
0.15	48.3	8.8%	0.914	0.019	0.021	0.021	7.5%	6.6%
0.20	85.0	14.5%	0.856	0.010	0.012	0.012	5.9%	5.8%

Notes: Simulation results with null causal effect $\beta_{X}=0$, and confounder effect $\beta_{U}=2$ to estimate the relative bias and empirical Type 1 error rate (5% nominal significance level) of the two‐stage least squares (or equivalently, inverse‐variance weighted) instrumental variable (IV) estimate; the relative bias is the bias of the IV estimate divided by the bias of the ordinary least squares (OLS) estimate. The relative bias is theoretically predicted to be close to the reciprocal of the mean value of the *F* statistic, (Mean *F*)^−1^.

Additional simulations are provided in Web Appendix A2. We varied the number of IVs (K=10) and considered different overlap proportions from 0% to 100% (Web Table A1). In this case, closer correspondence was observed between the expected and observed Type 1 error rates throughout, although these were across a narrower range. We also varied the sample size (N=1,000, Web Table A2), and considered the case of a binary risk factor (Web Table A3). In both these cases, there was close agreement between the expected and observed values of the relative bias and the Type 1 error rate. There was some deviation in Type 1 error estimates with a smaller sample size, with Type 1 error rates underestimated with stronger instruments (F parameter 5–10). However, this discrepancy appeared to be due to problems of maintaining nominal Type 1 error rates with weak instruments more generally rather than mis‐estimation of the relative bias, and would be resolved by using methods for inference that do not rely on the IV estimate having a normal distribution (for example, Fieller's theorem (Burgess, Small, & Thompson, 2015) or inversion of the Anderson–Rubin test statistic Mikusheva, 2010).

### 3.4 The *F* parameter and the *F* statistic

The bias of an IV estimate depends on the expected value of the *F* statistic (referred to here as the *F* parameter). However, in practice, the *F* parameter will be unknown and only the measured *F* statistic (an estimate of the *F* parameter) will be available in a single dataset. The *F* statistic can be highly variable. For example, we previously took a large study and divided it into 16 equally sized substudies at random (Burgess et al., 2011). By construction, each of these substudies should have had the same expected *F* statistic. However, the measured *F* statistics ranged from 3.4 to 22.6 (mean was 10.8).

One practical suggestion is to use an estimate of the *F* statistic based on an external dataset to ensure that the estimate of bias is not dependent on the *F* statistic in the data under analysis. This can be achieved by taking the value of *R* ^2^ (which is independent of sample size) from the external dataset and calculating the corresponding *F* statistic for the sample size under investigation; the *R* ^2^ for a single SNP is $2\;{\hat{\alpha }}^{2}\times MAF\times \left(1-MAF\right)$, where the genetic association with the risk factor $\hat{\alpha }$ is in standard deviation units, and MAF is the minor allele frequency. Additionally, the bias calculation can be repeated taking a lower value of the *F* statistic to address the problem that the *F* statistic in the data under analysis may be an overly optimistic estimate of the *F* parameter. For example, one could take the lower limit from a confidence interval for the *F* parameter (such as the lower limit of a one‐sided 95% confidence interval—only the lower tail of the interval is relevant). A method for constructing a confidence interval for the non‐centrality parameter of an *F* distribution (from which a confidence interval for the *F* parameter can be obtained) has been considered previously (Venables, 1975); this method is outlined in Web Appendix A3, and code for implementing the method is provided.

## 4 SIMULATION STUDY—BINARY OUTCOME

With a binary outcome in a case‐control setting (as is common for Mendelian randomization with a disease outcome), the ratio estimate is calculated by dividing the IV–outcome coefficient from logistic regression by the IV–risk factor coefficient from linear regression (Didelez, Meng, & Sheehan, 2010b). A two‐sample method can also be performed by replacing the linear model in the second‐stage regression of the outcome on the fitted values of the risk factor with a logistic model. When summarized data are available, a causal effect estimate can be obtained based using the IVW method to combine the ratio estimates, as in the case of a continuous outcome. There are some technical issues relating to the interpretation of these estimates with a binary outcome and a logistic regression model due to the non‐collapsibility of odds ratios (they approximate a population‐averaged log odds ratio per unit change in the distribution of the risk factor Burgess & CHD CRP Genetics Collaboration, 2013), but each is a consistent estimator under the null, and each provides a valid test of the null hypothesis of no causal effect (Vansteelandt, Bowden, Babanezhad, & Goetghebeur, 2011).

In principle, similar analytical formulae for bias under the null and Type 1 error rate could be developed with a binary outcome (Y=0,1). This would require a different formula for the variance of the IV estimate, as this depends on the number and ratio of participants with outcome events (Burgess, 2014):

(7) Variance  of  IV  estimate ( binary )≃1Nvar(X)ρ2P(Y=1)P(Y=0).

However, if the data on the outcome are derived from a case‐control sample, then typically the associations with the risk factor are estimated in control participants only (Bowden & Vansteelandt, 2011). This is for three main reasons: to avoid reverse causation, particularly if the risk factor is measured after the outcome event in cases; to avoid biases due to outcome‐dependent sampling (associations may be present in the case‐control sample even if they are absent in the underlying population) (Didelez, Kreiner, & Keiding, 2010); and because the controls are a more representative sample of the population as a whole (Didelez & Sheehan, 2007). Hence, even if there is overlap between datasets with a binary outcome, then provided that the IV–risk factor associations are estimated in the controls only, bias may not be substantial.

To investigate this, we simulated data using a similar data‐generating mechanism as previously:

(8) gik∼ Binomial (2,0.3) independently  for k=1,...,20xi=∑k=120αgik+ui+εXilogit(πi)=−3+βXxi+βUuiyi∼ Binomial (1,πi)ui∼N(0,1),εXi∼N(0,1) independently

A logistic‐linear relationship is assumed between the probability of an outcome event ($\pi_{i}$) and the risk factor and confounder. A case‐control sample was generated by simulating data on 100, 000 individuals, and taking the first 5,000 with an event ($y_{i}=1$) as cases, and the first 5,000 without an event ($y_{i}=0$) as controls. IV–outcome associations were estimated in all participants using logistic regression. The IV–risk factor associations were estimated both on the controls only, and in all participants (controls and cases). This was a one‐sample analysis (100% sample overlap). We set $\beta_{X}=0$ (null causal effect) and $\beta_{U}=1$, and considered scenarios with α=0.01,0.02,0.03,0.04,0.05,0.08.

### 4.1 Results

The mean estimates and empirical Type 1 (false positive) error rates based on 10, 000 simulated datasets are given in Table 3. With IV–risk factor associations estimated in the controls only, there was no detectable bias in the IV estimates even with extremely weak instruments, nor was there any inflation of Type 1 error rates. This suggests that a conventional Mendelian randomization analysis with a binary outcome in which the associations of the IV with the risk factor are only estimated in control participants provides a natural robustness against weak instrument bias, even in a one‐sample setting. With IV–risk factor associations estimated in all participants, bias was similar to that with a continuous outcome, with relative bias close to 1/E(F) on the log odds ratio scale and empirical Type 1 error rates close to the predicted values. However, the approximations were less accurate compared with the continuous outcome case, particularly for the weakest of instruments. This suggests that the same analytic formulae can be used with a binary outcome as with a continuous outcome, except with a different expression for the standard error of the IV estimate. R code for performing these calculations is given in Web Appendix A1.

**Table 3:** Simulation 3 with binary outcome to validate bias and type 1 error rate formulae

			Mean observational	Mean IV	Relative		Empirical	Expected
α	Mean *F*	Mean *R* ^2^	estimate	estimate	bias	(Mean *F*)^−1^	Type 1	Type 1
Risk factor measurements taken in controls only								
0.01	1.1	0.4%	0.481	0.001	0.002	‐	4.9%	‐
0.02	1.4	0.6%	0.481	0.000	0.000	‐	5.2%	‐
0.03	2.0	0.8%	0.479	−0.003	0.007	‐	4.8%	‐
0.04	2.7	1.1%	0.478	−0.001	−0.001	‐	5.0%	‐
0.05	3.7	1.5%	0.476	0.000	0.000	‐	5.2%	‐
0.08	7.9	3.1%	0.469	0.000	0.001	‐	4.7%	‐
Risk factor measurements taken in all participants								
0.01	1.2	0.3%	0.481	0.360	0.748	0.837	24.4%	29.1%
0.02	1.8	0.4%	0.481	0.237	0.493	0.561	17.4%	21.1%
0.03	2.8	0.5%	0.479	0.149	0.311	0.363	12.8%	15.2%
0.04	4.1	0.8%	0.478	0.099	0.207	0.242	10.0%	11.7%
0.05	5.9	1.2%	0.476	0.068	0.142	0.170	8.4%	9.6%
0.08	13.6	2.6%	0.469	0.030	0.064	0.074	6.3%	6.9%

Notes: Mean instrumental variable (IV) estimates and empirical Type 1 error rate (5% nominal significance level) from inverse‐variance weighted method with binary outcome for null causal effect ($\beta_{X}=0$) and six values of genetic associations with the risk factor (α) in a case‐control setting, with the risk factor measurements taken in control participants only and with the risk factor measurements taken in all participants. Observational estimates are log odds ratios from logistic regression of the outcome on the risk factor, and IV estimates are log odds ratios calculated using logistic regression for the IV–outcome association and linear regression for the IV–risk factor association.

## 5 EXAMPLES: SAMPLE OVERLAP BETWEEN LARGE CONSORTIA

We consider several Mendelian randomization analyses that could be undertaken using published summarized data from large consortia, and discuss the potential for bias due to participant overlap in each case.

1. **Body mass index and lipid traits**: We consider an analysis to estimate the causal effect of body mass index (BMI) on various lipid traits using data from the GIANT (Genetic Investigation of Anthropometric Traits) consortium (Locke et al., 2015) and the GLGC (Global Lipids Genetics Consortium) (The Global Lipids Genetics Consortium, 2013). 55 common studies are mentioned in the papers authored by these two consortia, comprising around 71% of participants in the GLGC. The 97 genetic variants reported as associated with BMI at a genome‐wide level of significance explain around 2.7% of the variance in BMI in the GIANT dataset (sample size = 339 224), corresponding to an *F* statistic of approximately $\frac{0.027}{1-0.027}\times \frac{339\;126}{97}=97.0$. The lower limit of a one‐sided 95% confidence interval for the F parameter is 93.7 (see Web Appendix A3 for calculation). Hence, despite the substantial overlap between the two consortia, considerable weak instrument bias would not be expected.
2. **Body mass index and coronary heart disease risk**: Although the *F* statistic suggests that weak instrument bias would not be substantial in any case, we consider the binary outcome of coronary heart disease (CHD), and investigate the degree of overlap between participants in the GIANT consortium above and the CARDIoGRAMplusC4D consortium (The CARDIoGRAMplusC4D Consortium, 2013). Of the 38 studies that appear in CARDIoGRAMplusC4D, 27 appear in GIANT in some form. In many cases, both case and control participants are included in GIANT. Hence, even though CHD is a binary outcome, the sample overlap between the two consortia could lead to weak instrument bias if the F parameter for BMI were lower.
3. **Educational attainment**: A genetic score for an individual's number of years of schooling (“EduYears”) constructed using five genetic variants associated with EduYears at a genome‐wide level of significance ($P<5\times 10^{-8}$) explained about 0.1% of the variance in EduYears in the discovery sample of 101, 069 individuals (corresponding *F* statistic = 20.2) (Rietveld et al., 2013). Associations were also reported for a follow‐up sample of 25, 490 individuals (corresponding *F* statistic = 5.1). The corresponding lower limits of the one‐sided 95% confidence intervals for the *F* parameter are 14.0 and 2.3 (see Web Appendix A3). Hence, a Mendelian randomization investigation using associations from the discovery sample should not lead to substantial weak instrument bias, but an investigation using associations from the follow‐up sample may be severely affected by weak instruments. However, if associations from the discovery sample are used in a Mendelian randomization investigation, then bias (in particular, selection bias) may be more serious due to winner's curse; this issues is explored further in the discussion.

These examples suggest that sample overlap between major international consortia may be substantial. Bias from weak instruments in very large consortia may not be substantial, but in moderately large consortia, potential bias, and inflated Type 1 error rates should be investigated.

## 6 DISCUSSION

In this paper, we have shown that bias in a Mendelian randomization investigation with a continuous outcome in a two‐sample setting is linearly related to the proportion of sample overlap between the two datasets. We have provided and validated analytical formulae for the expected bias and Type 1 error rate under the null given the F parameter (the expected value of the *F* statistic), the observational (OLS) estimate, the sample size, and the sample overlap percentage. With a binary outcome, provided that the IV–risk factor associations are estimated in the control participants only, bias due to sample overlap is negligible and Type 1 error rates are at nominal levels. If IV–risk factor associations are estimated in all participants, then bias is similar with a binary outcome (on the log odds ratio scale) as with a continuous outcome. Formulae for estimating the bias and Type 1 error rate under the null are given, and R code for calculating these quantities is given in Web Appendix A1. This code is implemented in a web application at https://sb452.shinyapps.io/overlap.

### 6.1 Overlap with datasets of different size

If the datasets are of different size, then the percentage overlap in these formulae should be taken with respect to the larger dataset, as this determines the correlation between the association estimates. This is because only individuals in both studies will lead to correlation between the association estimates, and additional individuals in either the association with the risk factor or with the outcome will attenuate any correlation. For example, if the smaller dataset has 1,000 participants, and the larger dataset has 10, 000 participants, then the sample overlap is only 10% even if all of the participants from the smaller dataset are included in the larger dataset.

### 6.2 Additional bias from genetic discovery

Weak instrument bias will be accentuated if the genetic variants were initially discovered in the data under analysis. This is due to winner's curse; if several genetic variants in truth have similar magnitudes of association with the risk factor, the association of the one that is the strongest in the data under analysis is likely to be overestimated (Burgess et al., 2011; Taylor et al., 2014). As this overestimation will generally mean that the associations with confounders are by chance stronger than expected, bias will occur if the discovery dataset is used in the estimation of the IV–risk factor or the IV–outcome associations. In a binary outcome setting, provided that only control participants were used in the discovery dataset, this should not lead to bias. However, if controls and cases were both used in the discovery dataset, this will lead to weak instrument bias.

There is no clear way to evaluate the bias due to overlap between the discovery sample and the dataset(s) used in a Mendelian randomization investigation. Hence, in such cases caution should be expressed, particularly if a genetic variant is close to the threshold statistical significance level for discovery or for inclusion in the Mendelian randomization analysis. In particular, data‐driven approaches for choosing genetic variants to be included in a Mendelian randomization analysis should be avoided. Analytical approaches to correct genetic associations for winner's curse may be useful in such a situation (Bowden & Dudbridge, 2009).

### 6.3 Increased bias in MR‐egger method

The recently introduced MR‐Egger method method has advantages over the conventional two‐stage least squares and inverse‐variance weighted methods in terms of some robustness to the instrumental variable assumptions being violated (Bowden, Davey Smith, & Burgess, 2015). Although weak instrument bias using data from large consortia may be low for conventional methods, bias for the MR‐Egger method has been shown to be considerably more pronounced, both attenuation in a two‐sample setting and bias toward the observational association in a one‐sample setting (Bowden, Davey Smith, Haycock, & Burgess, 2016a; Burgess, Bowden, Dudbridge, & Thompson, 2016a). This means that bias due to sample overlap may be more serious for the MR‐Egger method. In a two‐sample setting, bias in the MR‐Egger estimate does not depend on the proportion of variance explained by the IVs, but rather on the variability between the IV associations with the risk factor. Hence, for the MR‐Egger method, an I^2^ heterogeneity statistic is a better indicator of bias than the *F* statistic (Bowden et al., 2016). Further research is needed to derive an analytical formula for weak instrument bias in the MR‐Egger method in the one‐sample setting.

### 6.4 Practical recommendations

If there is sample overlap that is likely to lead to substantial bias and inflated Type 1 error rates in a “two‐sample” Mendelian randomization investigation, several approaches are available. If possible, the genetic associations with the risk factor could be derived from another non‐overlapping data source (possibly a subset of the original studies). A disadvantage of this is the potential loss of efficiency if the genetic associations are estimated less precisely (Burgess & Thompson, 2013). Alternatively, equal weights can be used, although again, there is a potential loss of power to detect a causal effect (Burgess et al., 2016b). (Software code for performing a summarized data analysis equivalent to a equally weighted allele score analysis is provided in Web Appendix A4.) Finally, sensitivity analyses can be performed using fewer but stronger genetic variants (and hence increasing the *F* parameter).

For consortia that publish genetic association estimates with continuous risk factors, we recommend that such estimates do not include case participants from case‐control studies. This will help reduce sample overlap in future Mendelian randomization studies that investigate the causal effect of the continuous risk factor on disease risk.

## Supporting information

Supporting Information

## References

[gepi21998-bib-0001] Angrist, J. , Imbens, G. , & Krueger, A. (1999). Jackknife instrumental variables estimation. Journal of Applied Econometrics, 14(1), 57–67.

[gepi21998-bib-0002] Angrist, J. , & Krueger, A. (1992). The effect of age at school entry on educational attainment: An application of instrumental variables with moments from two samples. Journal of the American Statistical Association, 87(418), 328–336.

[gepi21998-bib-0003] Angrist, J. , & Pischke, J. (2009). Mostly harmless econometrics: An empiricist's companion. Chapter 4: Instrumental variables in action: Sometimes you get what you need. Princeton: Princeton University Press.

[gepi21998-bib-0004] Angrist, J. D. , & Krueger, A. B. (1995). Split‐sample instrumental variables estimates of the return to schooling. Journal of Business and Economic Statistics, 13(2), 225–235.

[gepi21998-bib-0005] Bound, J. , Jaeger, D. , & Baker, R. (1995). Problems with instrumental variables estimation when the correlation between the instruments and the endogenous explanatory variable is weak. Journal of the American Statistical Association, 90(430), 443–450.

[gepi21998-bib-0006] Bowden, J. , Davey Smith, G. , & Burgess, S. (2015). Mendelian randomization with invalid instruments: Effect estimation and bias detection through Egger regression. International Journal of Epidemiology, 44(2), 512–525. 26050253 10.1093/ije/dyv080 PMC4469799

[gepi21998-bib-0007] Bowden, J. , Davey Smith, G. , Haycock, P. C. , & Burgess, S. (2016a). Consistent estimation in Mendelian randomization with some invalid instruments using a weighted median estimator. Genetic Epidemiology, 40(4), 304–314. 27061298 10.1002/gepi.21965 PMC4849733

[gepi21998-bib-0008] Bowden, J. , Del Greco, F. , Minelli, C. , Davey Smith, G. , Sheehan, N. A. , & Thompson, J. R. (2016b). Assessing the suitability of summary data for Mendelian randomization analyses using MR‐Egger regression: The role of the *I* ^2^ statistic. International Journal of Epidemiology, in press. 10.1093/ije/dyw220 PMC5446088 27616674

[gepi21998-bib-0009] Bowden, J. , & Dudbridge, F. (2009). Unbiased estimation of odds ratios: Combining genomewide association scans with replication studies. Genetic Epidemiology, 33(5), 406–418. 19140132 10.1002/gepi.20394 PMC2726957

[gepi21998-bib-0010] Bowden, J. , & Vansteelandt, S. (2011). Mendelian randomisation analysis of case‐control data using structural mean models. Statistics in Medicine, 30(6), 678–694. 21337362 10.1002/sim.4138

[gepi21998-bib-0011] Bun, M. J. , & Windmeijer, F. (2011). A comparison of bias approximations for the two‐stage least squares (2SLS) estimator. Economics Letters, 113(1), 76–79.

[gepi21998-bib-0012] Burgess, S. (2014). Sample size and power calculations in Mendelian randomization with a single instrumental variable and a binary outcome. International Journal of Epidemiology, 43(3), 922–929. 24608958 10.1093/ije/dyu005 PMC4052137

[gepi21998-bib-0013] Burgess, S. , Bowden, J. , Dudbridge, F. , & Thompson, S. G. (2016a). Robust instrumental variable methods using multiple candidate instruments with application to Mendelian randomization. *arXiv*, 1606.03729.

[gepi21998-bib-0014] Burgess, S. , Butterworth, A. S. , & Thompson, S. G. (2013). Mendelian randomization analysis with multiple genetic variants using summarized data. Genetic Epidemiology, 37(7), 658–665. 24114802 10.1002/gepi.21758 PMC4377079

[gepi21998-bib-0015] Burgess, S. , & CHD CRP Genetics Collaboration (2013). Identifying the odds ratio estimated by a two‐stage instrumental variable analysis with a logistic regression model. Statistics in Medicine, 32(27), 4726–4747. 23733419 10.1002/sim.5871 PMC3935453

[gepi21998-bib-0016] Burgess, S. , Dudbridge, F. , & Thompson, S. G. (2016b). Combining information on multiple instrumental variables in Mendelian randomization: Comparison of allele score and summarized data methods. Statistics in Medicine, 35(11), 1880–1906. 26661904 10.1002/sim.6835 PMC4832315

[gepi21998-bib-0017] Burgess, S. , Scott, R. , Timpson, N. , Davey Smith, G. , Thompson, S. G. , & EPIC‐InterAct Consortium (2015a). Using published data in Mendelian randomization: A blueprint for efficient identification of causal risk factors. European Journal of Epidemiology, 30(7), 543–552. 25773750 10.1007/s10654-015-0011-z PMC4516908

[gepi21998-bib-0018] Burgess, S. , Small, D. S. , & Thompson, S. G. (2015b). A review of instrumental variable estimators for Mendelian randomization. Statistical Methods in Medical Research, doi is 10.1177/0962280215597579. 10.1177/0962280215597579 PMC5642006 26282889

[gepi21998-bib-0019] Burgess, S. , & Thompson, S. G. (2011). Bias in causal estimates from Mendelian randomization studies with weak instruments. Statistics in Medicine, 30(11), 1312–1323. 21432888 10.1002/sim.4197

[gepi21998-bib-0020] Burgess, S. , & Thompson, S. G. (2013). Use of allele scores as instrumental variables for Mendelian randomization. International Journal of Epidemiology, 42(4), 1134–1144. 24062299 10.1093/ije/dyt093 PMC3780999

[gepi21998-bib-0021] Burgess, S. , & Thompson, S. G. (2015). Mendelian randomization: Methods for using genetic variants in causal estimation. Boca Raton, Florida, USA: Chapman & Hall.

[gepi21998-bib-0022] Burgess, S. , Thompson, S. G. , & CRP CHD Genetics Collaboration (2011). Avoiding bias from weak instruments in Mendelian randomization studies. International Journal of Epidemiology, 40(3), 755–764. 21414999 10.1093/ije/dyr036

[gepi21998-bib-0023] Cragg, J. , & Donald, S. (1993). Testing identifiability and specification in instrumental variable models. Econometric Theory, 9(02), 222–240.

[gepi21998-bib-0024] Dastani, Z. , Hivert, M.‐F. , Timpson, N. , Perry, J. R. B. , Yuan, X. , Scott, R. A. ,… Richards, J. B. (2012). Novel loci for adiponectin levels and their influence on type 2 diabetes and metabolic traits: A multi‐ethnic meta‐analysis of 45,891 individuals. PLOS Genetics, 8(3), e1002607. 22479202 10.1371/journal.pgen.1002607 PMC3315470

[gepi21998-bib-0025] Davey Smith, G. , & Ebrahim, S. (2003). ‘Mendelian randomization’: Can genetic epidemiology contribute to understanding environmental determinants of disease? International Journal of Epidemiology, 32(1), 1–22. 12689998 10.1093/ije/dyg070

[gepi21998-bib-0026] Davies, N. , von Hinke Kessler Scholder, S. , Farbmacher, H. , Burgess, S. , Windmeijer, F. , & Davey Smith, G. (2015). The many weak instrument problem and Mendelian randomization. Statistics in Medicine, 34(3), 454–468. 25382280 10.1002/sim.6358 PMC4305205

[gepi21998-bib-0027] Didelez, V. , Kreiner, S. , & Keiding, N. (2010a). Graphical models for inference under outcome‐dependent sampling. Statistical Science, 25(3), 368–387.

[gepi21998-bib-0028] Didelez, V. , Meng, S. , & Sheehan, N. (2010b). Assumptions of IV methods for observational epidemiology. Statistical Science, 25(1), 22–40.

[gepi21998-bib-0029] Didelez, V. , & Sheehan, N. (2007). Mendelian randomization as an instrumental variable approach to causal inference. Statistical Methods in Medical Research, 16(4), 309–330. 17715159 10.1177/0962280206077743

[gepi21998-bib-0030] Dobson, A. (2001). An introduction to generalized linear models. Boca Raton, Florida, USA: Chapman & Hall.

[gepi21998-bib-0031] Frost, C. , & Thompson, S. (2000). Correcting for regression dilution bias: Comparison of methods for a single predictor variable. Journal of the Royal Statistical Society: Series A (Statistics in Society), 163(2), 173–189.

[gepi21998-bib-0032] Greenland, S. (2000). An introduction to instrumental variables for epidemiologists. International Journal of Epidemiology, 29(4), 722–729. 10922351 10.1093/ije/29.4.722

[gepi21998-bib-0033] Hahn, J. , Hausman, J. , & Kuersteiner, G. (2004). Estimation with weak instruments: accuracy of higher‐order bias and MSE approximations. Econometrics Journal, 7(1), 272–306.

[gepi21998-bib-0034] Inoue, A. , & Solon, G. (2010). Two‐sample instrumental variables estimators. The Review of Economics and Statistics, 92(3), 557–561.

[gepi21998-bib-0035] Johnson, T. (2013). Efficient calculation for multi‐SNP genetic risk scores. Technical report, The Comprehensive R Archive Network. Retrieved from http://cran.r‐project.org/web/packages/gtx/vignettes/ashg2012.pdf

[gepi21998-bib-0036] Lawlor, D. , Harbord, R. , Sterne, J. , Timpson, N. , & Davey Smith, G. (2008). Mendelian randomization: Using genes as instruments for making causal inferences in epidemiology. Statistics in Medicine, 27(8), 1133–1163. 17886233 10.1002/sim.3034

[gepi21998-bib-0037] Locke, A. E. , Kahali, B. , Berndt, S. I. , et al. (2015). Genetic studies of body mass index yield new insights for obesity biology. Nature, 518(7538), 197–206. 25673413 10.1038/nature14177 PMC4382211

[gepi21998-bib-0038] Martens, E. , Pestman, W. , de Boer, A. , Belitser, S. , & Klungel, O. (2006). Instrumental variables: Application and limitations. Epidemiology, 17(3), 260–267. 16617274 10.1097/01.ede.0000215160.88317.cb

[gepi21998-bib-0039] Mikusheva, A. (2010). Robust confidence sets in the presence of weak instruments. Journal of Econometrics, 157(2), 236–247.

[gepi21998-bib-0040] Moreira, M. , Porter, J. , & Suarez, G. (2009). Bootstrap validity for the score test when instruments may be weak. Journal of Econometrics, 149(1), 52–64.

[gepi21998-bib-0041] Morris, A. , Voight, B. , Teslovich, T. , et al. (2012). Large‐scale association analysis provides insights into the genetic architecture and pathophysiology of type 2 diabetes. Nature Genetics, 44(9), 981–990. 22885922 10.1038/ng.2383 PMC3442244

[gepi21998-bib-0042] Nagar, A. (1959). The bias and moment matrix of the general *k*‐class estimators of the parameters in simultaneous equations. Econometrica: Journal of the Econometric Society, 27(4), 575–595.

[gepi21998-bib-0043] Nead, K. T. , Sharp, S. J. , Thompson, D. J. , et al. (2015). Evidence of a causal association between insulinemia and endometrial cancer: A Mendelian randomization analysis. Journal of the National Cancer Institute, 107(9), djv178. 26134033 10.1093/jnci/djv178 PMC4572886

[gepi21998-bib-0044] Nelson, C. , & Startz, R. (1990). The distribution of the instrumental variables estimator and its *t*‐ratio when the instrument is a poor one. Journal of Business, 63(1), 125–140.

[gepi21998-bib-0045] Pierce, B. , & Burgess, S. (2013). Efficient design for Mendelian randomization studies: Subsample and two‐sample instrumental variable estimators. American Journal of Epidemiology, 178(7), 1177–1184. 10.1093/aje/kwt084 PMC3783091 23863760

[gepi21998-bib-0046] Rietveld, C. A. , Medland, S. E. , Derringer, J. , et al. (2013). GWAS of 126,559 individuals identifies genetic variants associated with educational attainment. Science, 340(6139), 1467–1471. 23722424 10.1126/science.1235488 PMC3751588

[gepi21998-bib-0047] Staiger, D. , & Stock, J. (1997). Instrumental variables regression with weak instruments. Econometrica, 65(3), 557–586.

[gepi21998-bib-0048] Stock, J. , Wright, J. , & Yogo, M. (2002). A survey of weak instruments and weak identification in generalized method of moments. Journal of Business and Economic Statistics, 20(4), 518–529.

[gepi21998-bib-0049] Stock, J. , & Yogo, M. (2002). Testing for weak instruments in linear IV regression. SSRN eLibrary, 11, T0284.

[gepi21998-bib-0050] Sussman, J. , Wood, R. , & Hayward, R. (2010). An IV for the RCT: Using instrumental variables to adjust for treatment contamination in randomised controlled trials. British Medical Journal, 340, c2073. 20442226 10.1136/bmj.c2073 PMC3230230

[gepi21998-bib-0051] Taylor, A. , Davies, N. , Ware, J. , VanderWeele, T. , Davey Smith, G. , & Munafò, M. (2014). Mendelian randomization in health research: Using appropriate genetic variants and avoiding biased estimates. Economics and Human Biology, 13, 99–106. 24388127 10.1016/j.ehb.2013.12.002 PMC3989031

[gepi21998-bib-0052] The CARDIoGRAMplusC4D Consortium (2013). Large‐scale association analysis identifies new risk loci for coronary artery disease. Nature Genetics, 45(1), 25–33. 23202125 10.1038/ng.2480 PMC3679547

[gepi21998-bib-0053] The Global Lipids Genetics Consortium (2013). Discovery and refinement of loci associated with lipid levels. Nature Genetics, 45, 1274–1283. 24097068 10.1038/ng.2797 PMC3838666

[gepi21998-bib-0054] Vansteelandt, S. , Bowden, J. , Babanezhad, M. , & Goetghebeur, E. (2011). On instrumental variables estimation of causal odds ratios. Statistical Science, 26(3), 403–422.

[gepi21998-bib-0055] Venables, W. (1975). Calculation of confidence intervals for noncentrality parameters. Journal of the Royal Statistical Society: Series B (Methodological), 37(3), 406–412.

[gepi21998-bib-0056] Zhang, C. , Doherty, J. A. , Burgess, S. , et al. (2015). Genetic determinants of telomere length and risk of common cancers: A Mendelian randomization study. Human Molecular Genetics, 24(18), 5356–5366. 26138067 10.1093/hmg/ddv252 PMC4550826

