# Supplementary material for: Bias due to participant overlap in two‐sample Mendelian randomization
Source: Genet Epidemiol. 2016 Sep 14;40(7):597–608. doi: 10.1002/gepi.21998 (PMC5082560; doi:10.1002/gepi.21998)
Supplement: Supplementary file 1 — Supporting Information [file GEPI-40-597-s001.pdf]

Web Appendix

A1 Software code – estimation of bias

We provide R code to implement the formulae for the estimation of bias under the null and Type 1 error rate discussed in this paper. These formulae assume that there are multiple genetic variants; bias is a single genetic variant is unlikely to be substantial [Angrist and Pischke, 2009]. We assume that the units for the risk factor and outcome (for a continuous outcome) are the same for the ordinary least squares (OLS) and instrumental variable (IV) estimates; the simplest case is when all variables are in standard deviation units (so `var_x` and `var_y` are both 1).

```
expf = (N-K-1)/K * rsq/(1-rsq) # expf is expected value of the F statistic
                               # N is sample size
                               # K is number of genetic variants
                               # rsq is the expected value of R^2
                               # (otherwise expf can be specified directly)
bias = olsbias*overlap.prop*(1/expf)
                               # bias is the bias of the IV estimate under the null
                               # olsbias is the bias of the OLS estimate (observational
                               #  estimate for binary outcome)
                               # overlap.prop is the proportion of overlap
                               #  between the samples (between 0 and 1)
var  = var_y/(N*var_x*rsq)     # var is the variance of the IV estimate
                               # (continuous outcome)
                               # var_x is the variance of the risk factor
                               # var_y is the variance of the outcome
var  = 1/(N*rsq*var_x*prop.case*(1-prop.case))
                               # var is the variance of the IV estimate
                               # (binary outcome)
                               # prop.case is the proportion of cases (between 0 and 1)
type1err = 2-pnorm(1.96+bias/sqrt(var))-pnorm(1.96-bias/sqrt(var))
                               # type1err is the estimated Type 1 error rate
                               #  rate under the null for a nominal 5% two-sided
                               #  significance level
```

This code is implemented in a web application at `https://sb452.shinyapps.io/overlap`.

A2 Further validation of the analytic formulae for bias and Type 1 error rate

To further assess the validity of the analytic formulae for the bias and Type 1 error rate, we simulated data using the same data-generating model (2) under the causal null ($\beta_X = 0$) for a different level of confounding ($\beta_U = 1$), a different number of IVs ($K = 10$), and a different range of values of the IV strength ($\alpha = 0.02, 0.03, 0.05$) for cases where there is 0% overlap, increasing in increments of 10% up to a 100% overlap. In each case, we report the mean 2SLS (or equivalently IVW) estimate and empirical Type 1 error rate from 10 000 simulations, and the estimated bias and Type 1 error rate from the analytic formulae above, using mean values of the OLS estimate and F statistic estimated across the simulations (the relative bias is estimated as the reciprocal of the average value of the F statistic across simulations – this would not be available for a single dataset in practice).

Results are given in Table A1. The Monte Carlo standard error for the observed mean estimate is around 0.002, and for the Type 1 error rate is around 0.2 to 0.3%. We see that the analytical formulae slightly overestimate the observed bias and Type 1 error inflation. However, the estimated bias and Type 1 error rate are close to the observed values throughout.

	$\alpha = 0.02$		$\alpha = 0.03$		$\alpha = 0.05$	
	Mean $F = 1.8$		Mean $F = 2.9$		Mean $F = 6.3$	
	Mean OLS = 0.500		Mean OLS = 0.499		Mean OLS = 0.497	
Percentage overlap	Expected Bias (Error)	Observed Mean (Error)	Expected Bias (Error)	Observed Mean (Error)	Expected Bias (Error)	Observed Mean (Error)
0%	0.000 (5.0)	-0.002 (4.9)	0.000 (5.0)	0.002 (5.0)	0.000 (5.0)	-0.002 (5.2)
10%	0.027 (5.2)	0.024 (4.9)	0.017 (5.1)	0.018 (5.3)	0.008 (5.0)	0.005 (5.1)
20%	0.054 (5.6)	0.049 (5.5)	0.035 (5.4)	0.034 (5.2)	0.016 (5.2)	0.012 (5.2)
30%	0.082 (6.4)	0.076 (6.2)	0.052 (5.9)	0.049 (5.8)	0.024 (5.4)	0.018 (5.4)
40%	0.109 (7.5)	0.103 (7.4)	0.069 (6.6)	0.064 (6.8)	0.032 (5.7)	0.026 (5.5)
50%	0.136 (9.0)	0.128 (8.2)	0.086 (7.5)	0.079 (7.2)	0.040 (6.1)	0.033 (5.7)
60%	0.163 (10.8)	0.153 (9.6)	0.104 (8.6)	0.096 (7.9)	0.048 (6.6)	0.040 (6.1)
70%	0.191 (12.9)	0.178 (11.3)	0.121 (10.0)	0.111 (9.1)	0.056 (7.2)	0.047 (6.6)
80%	0.218 (15.4)	0.205 (13.7)	0.138 (11.5)	0.126 (10.6)	0.064 (7.9)	0.055 (6.8)
90%	0.245 (18.3)	0.231 (16.3)	0.156 (13.3)	0.143 (12.2)	0.072 (8.7)	0.063 (7.8)
100%	0.272 (21.4)	0.258 (18.3)	0.173 (15.3)	0.159 (13.5)	0.079 (9.6)	0.068 (8.4)

Web Table A1: Comparison of the expected bias with a null causal effect ($\beta_X = 0$) and Type 1 error rate (5% significance level) calculated using formulae (3) and (4) with the observed mean and empirical Type 1 error rate from a simulation study for a two-sample IV estimate with sample overlap and 10 IVs.

We also repeated the simulation of Table II, except with a sample size of 1000 rather than 10 000. Results for $\alpha = 0.01, 0.02, 0.03$ have been omitted, as the instruments are so weak that they are barely associated with the risk factor (even an F statistic of 1.3 corresponds to a two-sided p-value of 0.34 for an F distribution on 20 and 979 degrees of freedom, and an R^2 statistic of 2.7% for a sample size of 1000 is little more than would be explained on average by a variable that was truly independent from the risk factor), and so would not be used as instruments in practice. Generally the expected estimates of relative bias (reciprocal of F parameter) are close to the observed values. However, while Type 1 error rate is overestimated for the weakest of instruments, it is underestimated for large values of α. This may be due to overrejection of the null by the two-stage least squares method using a Wald test with weak instruments [Stock and Yogo, 2002]. There are several solutions to this problem, including the use of Fieller's theorem [Burgess et al., 2015b], inversion of the Anderson–Rubin test statistic [Mikusheva, 2010], and bootstrapping [Moreira et al., 2009] for improved inference properties with weak instruments; none of these methods assume that the IV estimate is normally distributed.

Web Table A2: **Repeat of Simulation 2 with continuous outcome to validate bias and Type 1 error rate formulae with reduced sample size**

α	Mean F	Mean R^2	Mean OLS estimate	Mean IV estimate	Relative bias	(Mean $F)^{-1}$	Empirical Type 1 error	Expected Type 1 error
0.04	1.3	2.7%	0.994	0.747	0.751	0.743	80.6%	84.9%
0.05	1.5	3.0%	0.989	0.651	0.658	0.657	75.0%	78.9%
0.06	1.8	3.5%	0.986	0.560	0.568	0.570	67.7%	71.0%
0.07	2.0	4.0%	0.980	0.484	0.494	0.490	62.5%	62.4%
0.08	2.3	4.5%	0.974	0.418	0.429	0.428	54.9%	54.6%
0.09	2.7	5.2%	0.967	0.355	0.367	0.369	48.6%	46.7%
0.10	3.1	6.0%	0.959	0.306	0.319	0.321	42.3%	40.2%
0.15	5.7	10.5%	0.913	0.161	0.177	0.174	24.5%	20.8%
0.20	9.4	16.1%	0.856	0.098	0.115	0.106	17.2%	13.0%

Simulation results with null causal effect $\beta_X = 0$, and confounder effect $\beta_U = 2$ to estimate the relative bias and empirical Type 1 error rate (5% nominal significance level) of the two-stage least squares (or equivalently, inverse-variance weighted) instrumental variable (IV) estimate; the relative bias is the bias of the IV estimate divided by the bias of the ordinary least squares (OLS) estimate. The relative bias is theoretically predicted to be close to the reciprocal of the mean value of the F statistic, (Mean $F)^{-1}$. This table is a repeat of Table II from the main paper, except with a smaller sample size.

We also repeated the simulation of Table II, except with a binary risk factor. This was implemented by first simulating the risk factor as in equation (2), and then dichotomizing the risk factor at its median to take the value 0 or 1. Associations with the risk factor were still estimated using a linear model in the two-stage least squares method. Web Table A3 shows marked differences in the mean OLS and IV estimates compared with those in Table II; however, the expected estimates of relative bias and Type 1 error rate were close to their observed values for all strengths of instrument.

Web Table A3: **Repeat of Simulation 2 with continuous outcome to validate bias and Type 1 error rate formulae with binary risk factor**

α	Mean F	Mean R^2	Mean OLS estimate	Mean IV estimate	Relative bias	(Mean $F)^{-1}$	Empirical Type 1 error	Expected Type 1 error
0.01	1.1	0.2%	2.256	1.995	0.884	0.882	66.6%	66.3%
0.02	1.5	0.3%	2.254	1.449	0.643	0.654	52.8%	52.1%
0.03	2.2	0.4%	2.252	1.004	0.446	0.455	37.9%	39.4%
0.04	3.1	0.6%	2.249	0.687	0.305	0.318	27.0%	28.5%
0.05	4.3	0.9%	2.245	0.494	0.220	0.231	20.3%	22.0%
0.06	5.8	1.1%	2.240	0.368	0.164	0.172	16.0%	17.9%
0.07	7.5	1.5%	2.234	0.280	0.125	0.133	13.2%	14.9%
0.08	9.5	1.9%	2.226	0.217	0.097	0.106	11.3%	11.8%
0.09	11.7	2.3%	2.219	0.176	0.079	0.086	10.0%	10.7%
0.10	14.2	2.8%	2.211	0.142	0.064	0.071	9.0%	9.7%
0.15	30.1	5.7%	2.157	0.071	0.033	0.033	6.7%	7.5%
0.20	51.4	9.3%	2.087	0.038	0.018	0.019	5.9%	5.9%

Simulation results with null causal effect $\beta_X = 0$, and confounder effect $\beta_U = 2$ to estimate the relative bias and empirical Type 1 error rate (5% nominal significance level) of the two-stage least squares (or equivalently, inverse-variance weighted) instrumental variable (IV) estimate; the relative bias is the bias of the IV estimate divided by the bias of the ordinary least squares (OLS) estimate. The relative bias is theoretically predicted to be close to the reciprocal of the mean value of the F statistic, (Mean $F)^{-1}$. This table is a repeat of Table II from the main paper, except with a binary risk factor.

A3 Software code – bound for the F parameter

The lower bound of a one-sided 95% confidence interval for the F parameter can be obtained by: i) finding the non-centrality parameter for which the given value of the F statistic is the mean of the non-central F distribution; ii) finding the value such that the cumulative distribution function for that non-central F distribution is 5%.

We demonstrate this procedure with the example from the paper of body mass index in which the F statistic is 97.02, and the degrees of freedom for the F distribution are $\nu_1 = 97$ and $\nu_2 = 339\,127$. The mean of the non-central F distribution on (ν_1, ν_2) degrees of freedom with non-centrality parameter λ (for $\nu_2 > 2$) is:

$$\mathbb{E}(F) = \frac{\nu_2(\nu_1 + \lambda)}{\nu_1(\nu_2 - 2)}.$$

In this case, the non-centrality parameter is:

$$\lambda = \frac{97 \times 97.02 \times 339\,125}{339\,127} - 97 = 9313.88$$

The cumulative distribution function of the non-central F distribution can be calculated using the R code:

```
pf(value, df1=97, df2=339127, ncp=9313.884)
```

We find the value such that the distribution function takes the value 0.05 by an iterative search:

```
> pf(94, df1=97, df2=339127, ncp=9313.884)
[1] 0.06549997
> pf(93, df1=97, df2=339127, ncp=9313.884)
[1] 0.02182131
> pf(93.5, df1=97, df2=339127, ncp=9313.884)
[1] 0.03890898
> pf(93.75, df1=97, df2=339127, ncp=9313.884)
[1] 0.05084209
> pf(93.625, df1=97, df2=339127, ncp=9313.884)
[1] 0.04455657
> pf(93.6875, df1=97, df2=339127, ncp=9313.884)
[1] 0.04761685
> pf(93.71875, df1=97, df2=339127, ncp=9313.884)
[1] 0.04920851
> pf(93.734375, df1=97, df2=339127, ncp=9313.884)
[1] 0.05002001
```

Hence the lower limit of the one-sided 95% confidence interval is approximately 93.734.

The following function performs this search automatically:

```
findlowerflimit <- function(f, nu1, nu2) {
 lambda = f*nu1*(nu2-2)/nu2-nu1
 lower = f-1
 while (pf(lower, df1=nu1, df2=nu2, ncp=lambda)>0.05) { lower = lower-1 }
 upper = lower + 1
 while ( abs(pf((lower+upper)/2, df1=nu1, df2=nu2, ncp=lambda)-0.05)>0.0001 ) {
 if (pf((lower+upper)/2, df1=nu1, df2=nu2, ncp=lambda)>0.05)
    { upper = (lower+upper)/2 }
 if (pf((lower+upper)/2, df1=nu1, df2=nu2, ncp=lambda)<0.05)
    { lower = (lower+upper)/2 }
 }
 return((lower+upper)/2)
 }
```

Running this code gives:

```
> findlowerflimit(97.02, 97, 339127)
[1] 93.73484
```

Hence the lower limit of the one-sided 95% confidence interval is approximately 93.735. Further precision can be obtained if required, but 1 decimal place should be enough to give a good estimate of the potential bias.

For comparison, the corresponding lower limits for educational attainment are:

1. Discovery sample: observed F statistic = 20.2, sample size = 101 069, number of genetic variants = 5, lower limit for F statistic = 14.0.

2. Follow-up sample: observed F statistic = 5.1, sample size = 25 490, number of genetic variants = 5, lower limit for F statistic = 2.3.

In these cases, the lower one-sided 95% limit for the F statistic is substantially lower than the observed value of the F statistic.

A4 Software code – allele score method using summarized data with arbitrary weights

The estimate from an inverse-variance weighted method for uncorrelated genetic variants is equal to that from of an allele score method with internally-derived weights (also equal to that from a two-stage least squares method). The inverse-variance weighted method can be modified to give the same estimate as that from an allele score method with arbitrary weights [Burgess et al., 2016b]. In particular, it can provide the same estimate as that from an allele score method with equal weights.

Inputs are the genetic associations with the risk factor (`bx`, standard error `bxse`), the genetic associations with the outcome (`by`, standard error `byse`), and the allele score weights (`wts`):

```
wts      = rep(1, length(bx))
   # for equal weights, otherwise specify weights
beta_SSw = sum(wts*by/byse^2)/sum(wts*bx/byse^2)
   # equivalent to original IVW method when wts = bx
se_SSw_first   = sqrt(sum(wts^2/byse^2)/sum(wts*bx/byse^2))
   # standard error from delta method (first-order approximation)
se_SSw_second  = sqrt(sum(wts^2/byse^2)/sum(wts*bx/byse^2)^2 +
  sum(wts*by/byse^2)^2/sum(wts*bx/byse^2)^4*sum(wts^2/byse^2) -
  2*theta*sum(wts*by/byse^2)/sum(wts*bx/byse^2)^3)
   # standard error from delta method (second-order approximation)
   # theta is the correlation between the numerator and denominator of the estimate
   # if the correlation is not known, it can be taken as the observational
   # correlation between the risk factor and outcome;
   # a sensitivity analysis can also be performed for its value
```

If the genetic variants are correlated, then a valid test statistic for the causal null hypothesis can be derived:

```
beta_SSw_cor = sum(wts*by/byse^2)/sum(wts^2/byse^2)
se_SSt_cor   = sqrt(sum((wts*byse^-1)%o%(wts*byse^-1)*rho)/
  sum(wts^2/byse^2)^2)
   # standard error from delta method (first-order approximation)
   # rho is the matrix of correlations between the genetic variants
```

This test statistic can also be used with uncorrelated genetic variants [Burgess et al., 2016b], although it does not have an interpretation as an effect estimate with either correlated or uncorrelated variants.
